# Influence of Education on Oro-dental Knowledge among School Hygiene Instructors

**DOI:** 10.5681/joddd.2009.013

**Published:** 2009-06-05

**Authors:** Soussan Irani, Marjane Meschi, Azizollah Goodarzi

**Affiliations:** ^1^Assistant Professor, Department of Oral Pathology, Faculty of Dentistry, Hamadan University of Medical Sciences, Hamadan, Iran; ^2^PhD student, Department of Community Health, Faculty of Dentistry, Hamadan University of Medical Sciences, Hamadan, Iran; ^3^DDS, Private Practice, Hamadan, Iran

**Keywords:** Health care instructor, education, oro-dental health

## Abstract

**Background and aims:**

Recent progresses in preventive dentistry and their correct application in many developed coun-tries have remarkably decreased the rate of oro-dental diseases in children and teenagers, while the rate of oro-dental diseases is on the rise among the children in developing countries. The aim of this study was to evaluate the impact of educating school health care instructors by measuring their level of oral health knowledge and their opinions about the impact of oral health and preventive dentistry.

**Materials and methods:**

This was a cross-sectional descriptive-analytical study. Questionnaires were administered before and after an educational lecture to school health care instructors in Hamadan, Iran. Data were analyzed using paired t-test.

**Results:**

In this study, 31 school health care instructors took part. The percentage of instructors in poor knowledge level was 22.6% before the educational lecture (education), which decreased to 0 percent after the education (P < 0.05). The percentage of instructors with good knowledge level was 3.2%, which increased to 80.6% after the education (P < 0.05).

**Conclusion:**

Close cooperation between universities and the Ministry of Health and Medical Education will lead to im-provements in the level of knowledge and awareness of school health care instructors.

## Introduction

Health promotion is the process of enabling peo-ple to increase control over, and to improve, their health. To reach a state of complete physical, mental and social well-being an individual or group must be able to identify and to realize aspirations, to satisfy needs, and to change or cope with the envi-ronment. Health is, therefore, seen as a resource for everyday life, not the aim of living. Health is a posi-tive concept emphasizing social and personal re-sources, as well as physical capacities. Therefore, health promotion is not just the responsibility of the health sector, but goes beyond healthy lifestyles to well-being. ^1^

Tooth decay is a problem in young children and is aggravated due to existing barriers that prevent them from obtaining dental care. The availability of effective preventive methods improves dental treatment so that children no longer suffer from dental problems.^2^

At present, common oro-dental diseases such as dental caries and gingivo-periodontal diseases are considered health-threatening diseases. The prevalence of childhood caries is a public health problem. According to statistics, 61% of 6-12-year-old children have at least one tooth cavity, and/or filling in their deciduous teeth, and 40% of 6-14-year old individuals have at least one cavity and/or filling in their permanent teeth. These diseases have a great effect on the economy of a society due to high prevalence in all social classes.^3^

In Iran, DMFT indices for 6-, 9- and 12-year-old children are 0.2, 0.9 and 1.5, respectively; and dmft indices for 3-, 6- and 9-year-old children are 1.7, 4.8 and 3.3, respectively.^4^

Recent progresses in preventive dentistry and their correct application in many developed countries have resulted in marked decrease in the rate of oro-dental diseases in children and teenagers, while the rate of oro-dental diseases is on the rise among children in developing countries.^5^

Dental caries in primary dentition not only shows children’s oral health, but also predicts the probability of caries in permanent dentition and general health status.^6^

Many investigations have shown the association between periodontal diseases and systemic diseases, including coronary heart disease and diabetes.^7^

Children with caries have a slower growth rate compared with children without it, which can be attributed to the pain during eating.^6^

Oral health promotion focuses largely on disease, and health is defined as the absence of caries and periodontitis. Oral health education aims to impart knowledge to people and influence their choice of lifestyle.^8^

Oral health education for children should be considered a priority. School instructors play an important role in achieving the best oral health outcomes for school children because in some areas children have limited access to dental care and the school instructors are the first health professionals to come in contact with children. Considering the “important role” of school instructors, it is essential to explore their knowledge.

This study was conducted to evaluate the impact of educating school health care instructors with the aim of taking a step toward enhancing preventive policy in oro-dental hygiene by measuring their level of oral health knowledge and their opinions about the impact of oral health and preventive dentistry.

## Materials and Methods

A cross-sectional descriptive-analytical study was undertaken among school health care instructors. All the school health instructors of the City of Hamadan were invited to the Faculty of Dentistry but only 31 school health care instructors participated in this study. A questionnaire which included 40 multiple-choice questions and two multiple-choice answer sheets were developed and given to participants, one before the educational lecture (as pre-test) and one immediately after the lecture as post-test. The lecture and the tests which took 2 hours focused on  tooth anatomy and biology, chronology of primary and permanent  dentition and also shedding time of primary teeth, the impact of dental care, various preventive strategies of dental health care, and the role of diet and fluoride therapy. Then all the answer sheets were collected. Data were analyzed using SPSS, Version 12, and paired t-test.

## Results

In this study, 31 school health care instructors participated. They had experience of 5 to 15 years with a mean of 12.5 years.

**Figure 1:**
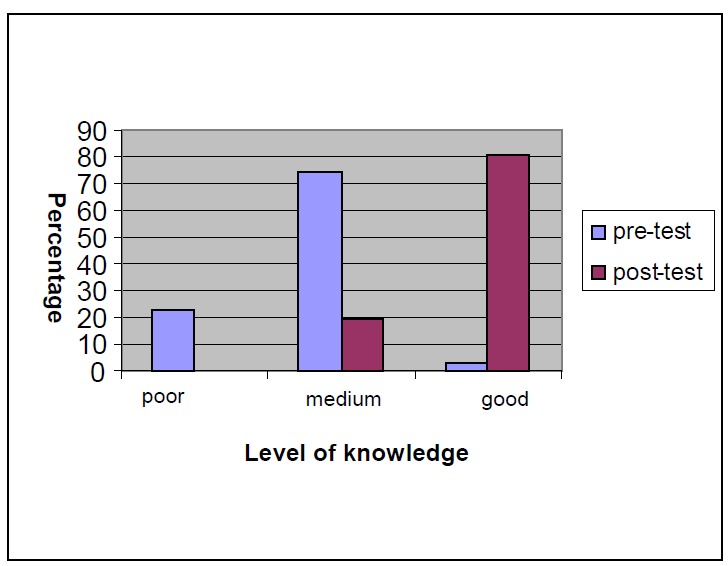
Comparison of the level of knowledge of school health care instructors before and after the educational lecture.

Figure 1 compares the level of knowledge of school health care instructors before and after the educational lecture. As seen in Figure 1, the score less than 20 was considered poor knowledge level, the score between 20 and 31 was considered medium knowledge level and a score higher than 31 was considered good knowledge level. Regarding this profile, the percentage of instructors in poor knowledge level was 22.6% before the educational lecture, which decreased to 0 percent after the educational lecture (P < 0.05). In addition, the percentage of individuals in the medium knowledge level was 74.2% and 19.4 % before and after education, respectively (P < 0.05). The percentage of instructors with good knowledge level was 3.2%, which increased to 80.6% after the education (P < 0.05), demonstrating a statistically significant difference (P < 0.05).

Table 1 demonstrates comparison of the scores of knowledge of school health care instructors in Hamadan before and after the education.

**Table 1:** Comparison of the scores of knowledge of school health care instructors before and after the education

score of knowledge before education (pre-test) Mean ± SD	score of knowledge after ducation (post-test) Mean ± SD	Number	T	P value
23.83 ± 4.73	33.19 ± 3.2	31	11.63	0.000

Paired t-test

The scores of knowledge of school health care instructors before (pre-test) and after education (post-test) were 23.83 ± 4.73 and 33.19 ± 3.2, respectively (t = 11.63; P = 0.000).

## Discussion

Tooth decay and periodontal diseases are gradually increasing in children, emphasizing the necessity of educating school health care instructors as the first step in preventing oro-dental diseases. Oral diseases are both individual and community health problems.^5^

Preventive education of dental care must enhance the opportunity for a life free of oral diseases.This research study evaluated the effectiveness of an educational program for 31 school health care instructors in Hamadan on the basis of pre-test and post-test answer sheets. The results showed that educating school health care instructors has a great influence on improving their knowledge about oro-dental health.

Remarkable improvements were seen in adults after performing educational services in oral hygiene in one survey.^9^ It was also found that children under 5 have a capacity for learning these subjects as well.^9^

According to Simmons et al,^10^ most children aged 2-4 are not able to understand these concepts, but education is useful for pre-school children.

In a research study in Yazd City, Iran, knowledge of 72 school health care instructors was evaluated through a questionnaire which included 7 questions about their basic knowledge about oral health and 20 questions about their awareness. The following results were achieved: The rate of general knowledge in younger instructors with fewer years of experience was more than the instructors with more experience, which might be attributed to recent graduation of younger instructors from the university.^11^

In another study in Hamadan,^12^ the following results were achieved: More than 77% of Hamadan school health care instructors have worked for 15 years. The average of scores of their knowledge was 16.03, which might be considered a medium score. School health care instructors with up to 15 years of experience had a similar situation, consistent with the results of the present study.

## Conclusion

Based on the results of this study the following conclusions were arrived at:

1. School health instructors need to improve their knowledge to reduce dental caries prevalence among schoolchildren. Therefore, education of school instructors should be programmed by the authorities. Close cooperation between universities and Ministry of Health and Medical Education will give rise to improvements in the level of knowledge and awareness of school health care instructors.
2. Because one of the most important determinants of dental caries is low socioeconomic status, preventive dentistry programs could result in a higher level of oral health.
3. Public insurance provides limited coverage for oral health; therefore, for better oral health care it is necessary to design and introduce a new insurance system, covering all types of dental treatments. Therefore, it is recommended that governmental insurance should cover dental treatments.
4. The government should also provide screening services for early detection of dental diseases. As a result, coordination of services between schools and dental profession is highly recommended
5. Training of general dental practitioners for the treatment of young children is another way to promote dental health in children.
